# Nematomorph parasites potentially drive nutritional flow from terrestrial to aquatic ecosystems

**DOI:** 10.1093/pnasnexus/pgag201

**Published:** 2026-07-07

**Authors:** Ayano Medo, Takuya Sato

**Affiliations:** Center for Ecological Research, Kyoto University, Hirano, Otsu, Shiga 520-2113, Japan; Center for Ecological Research, Kyoto University, Hirano, Otsu, Shiga 520-2113, Japan; KYOUSEI Science Center for Life and Nature, Nara Women's University, Hirano, Otsu, Shiga 520-2113, Japan

**Keywords:** nutrient flux, EPA, extended phenotype, host manipulation, ecosystem linkage

## Abstract

Long-chain polyunsaturated fatty acids (LC-PUFA), particularly eicosapentaenoic acid (EPA; 20:5n-3) are synthesized primarily by algae, such as diatoms and cryptophytes, in aquatic ecosystems. Although LC-PUFA flux has commonly been considered unidirectional from aquatic to terrestrial ecosystems, recent evidence suggests a potential reciprocal flow of EPA from terrestrial to aquatic systems via amphibious insects and terrestrial arthropods. Here, we report the possibility that nematomorph parasites drive the flow of EPA from terrestrial to aquatic ecosystems by inducing EPA-bearing terrestrial hosts to jump into streams. Individuals of camel crickets, a major host of the nematomorphs, had ∼4–17 times higher EPA mass than those of known aquatic invertebrates. An endangered charr population acquired more EPA from the camel crickets than from aquatic invertebrates in late summer through autumn, when the nematomorphs manipulated their hosts, in a temperate stream. Our results suggest that host manipulation by parasites potentially drives essential organic nutrient flow, with particular emphasis on the need to recognize reciprocal PUFA flow between terrestrial and aquatic ecosystems.

## Introduction

Food quality can have a more direct effect than food quantity on the growth and reproduction of wildlife, and ultimately on their population dynamics and adaptive evolution (1, 2). The amount and composition of organic compounds such as amino acids, vitamins, and fatty acids available in ecosystems determine the food quality (3). However, the complex nature of the synthesis, accumulation, and flow of these organic compounds within and across ecosystems is less understood than that of the inorganic nutrients and energy that regulate food quantities (1).

Long-chain polyunsaturated fatty acids (LC-PUFAs), particularly eicosapentaenoic acid (EPA; 20:5n-3) and docosahexaenoic acid (22:6n-3), are synthesized primarily by algae, such as diatoms and cryptophytes, in aquatic ecosystems (2, 4). LC-PUFA fluxes have generally been viewed as dominated by transfers from aquatic to terrestrial ecosystems, such as through the emergence of aquatic insects accumulating algal EPA (1, 4). In contrast, recent studies suggest a potential reciprocal flow of EPA from terrestrial to aquatic ecosystems via amphibious insects and terrestrial arthropods (5). However, quantitative comparisons among multiple EPA flux pathways within and across ecosystems remain limited, obscuring a comprehensive understanding of PUFA fluxes in nature.

**Figure 1:**
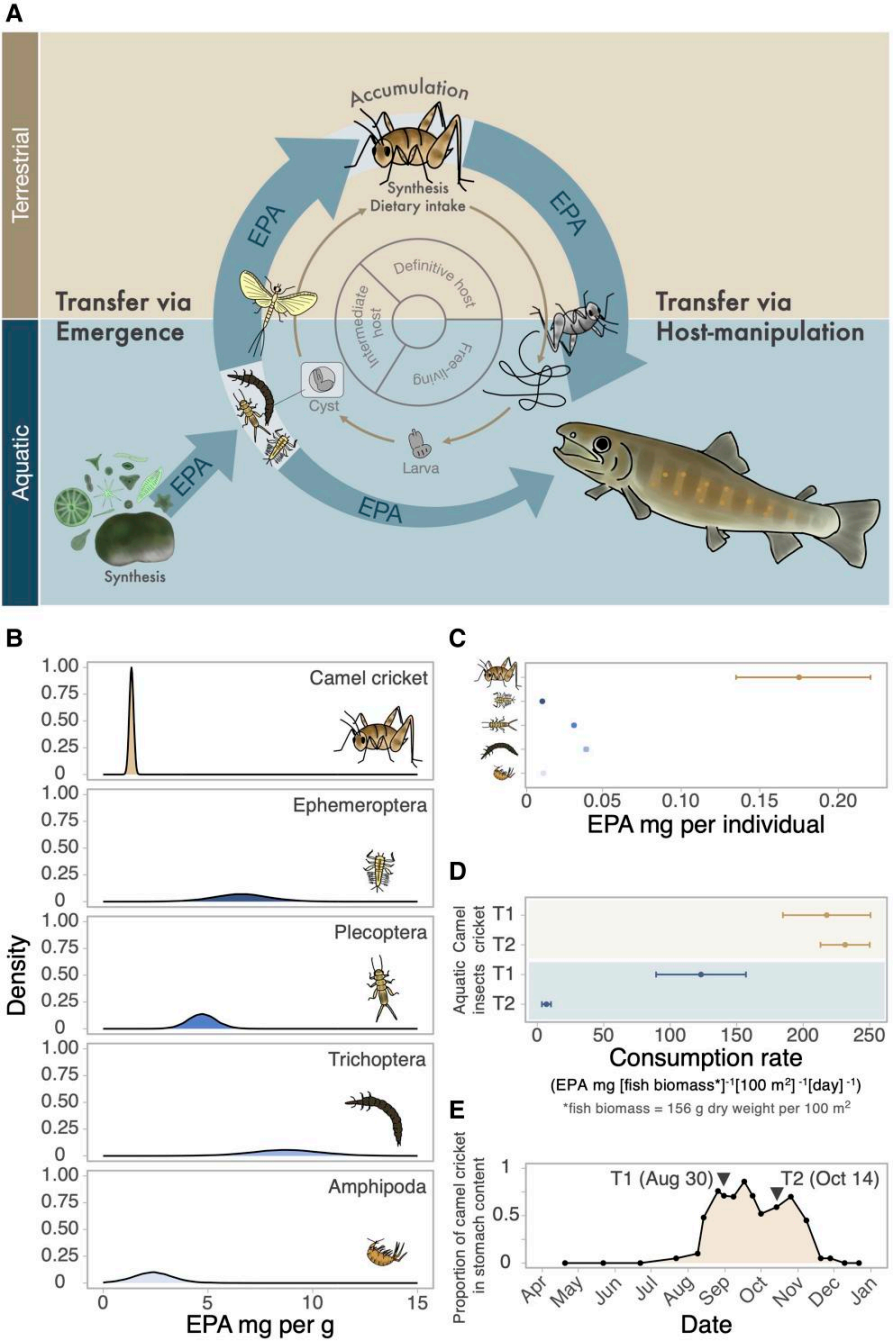
Schematic overview of the hypothesized reciprocal EPA flow between forest and stream ecosystems, taxon-specific EPA contents, and EPA intake of fish. A) Hypothesized pathways of EPA (20:5n-3) and life cycle of the nematomorph parasite underlying the EPA flow. B) Posterior distributions of EPA content for camel crickets, a major host of nematomorph parasites, and each order of aquatic invertebrates, including Ephemeroptera, Plecoptera, Trichoptera, and Amphipoda. C) Estimated EPA content per individual prey ingested by fish consumers for camel crickets and aquatic invertebrates, including Ephemeroptera, Plecoptera, Trichoptera, and Amphipoda. In the panel, data points and error bars indicate the mean and 95% credible interval of the estimated EPA content per individual prey. D) Estimated daily area-based EPA intake of fish from camel crickets and aquatic invertebrates. T1 and T2 represent the two sampling dates during the host manipulation by nematomorphs. E) Seasonal variation in the proportion of fish that ingested camel crickets. Points represent sampling dates, and T1 and T2 are consistent with those in Fig. 1D.

Nematomorph parasites induce their terrestrial arthropod hosts, such as crickets and ground beetles, to enter water, where the parasites must emerge to reproduce (6). Stream salmonids readily eat these infected arthropods which, due to their large body size and abundance, accounted for 60% of the annual energy intake of an endangered charr population in a temperate forested stream (6). However, it remains unknown whether these hosts can also contribute to the transfer of essential organic compounds from terrestrial to aquatic ecosystems. If camel crickets contain measurable amounts of EPA, which often harbor nematomorphs, nematomorph-induced host manipulation may drive the flow of EPA from terrestrial to stream ecosystems (Fig. 1A). Quantifying the EPA flow pathways to stream fish is therefore essential for testing the reciprocal flow of EPA between forest and stream ecosystems.

## Results and discussion

We first quantified EPA contents (mg g^−1^ in dry weight) and the mass (mg) per individual camel cricket (*Diestrammena tsushimensis* and *Diestrammena itoda*), a major host of the nematomorphs and scavenger for weakened and dead aquatic insects (7), in a temperate stream in Japan. In addition, EPA contents of aquatic invertebrates were estimated by the meta-analysis. The relative EPA amount (%) was higher in aquatic invertebrates than in camel crickets. However, the crickets had EPA contents (1.35 mg g^−1^) comparable to an aquatic amphipod (2.36 mg g^−1^; Fig. 1B). Given ∼20–74 times larger body weight of camel crickets relative to aquatic invertebrates, individuals of camel crickets had ∼4–17 times more EPA amount than those of known aquatic invertebrates (0.17 vs. 0.01–0.04 mg per individual; Fig. 1C). The relative EPA content of camel crickets was 3.71 ± 1.68% of total fatty acids (mean ± SD, *n* = 10; Supplemental Information, SI) and did not differ significantly between infected and uninfected crickets. In addition, EPA as well as other n-3 PUFA were below the detection limit in nematomorphs (SI), indicating that nematomorph infections did not reduce the EPA content of the camel crickets. The EPA content of the crickets was lower than those of the aquatic insects (10–15%), although it would be sufficient to support the physiological functions of stream-dwelling salmonid fish, as the relative EPA contents of artificial food for farmed salmonid fish is ∼2–3%. However, the low digestibility of terrestrial invertebrates by stream-dwelling salmonids might lead to low accumulation of LC-PUFA in their body tissues (8). Compound-specific stable isotope analyses (CSIA) could provide insight into the assimilation of LC-PUFA from terrestrial invertebrates by stream-dwelling fish.

We next quantified the potential flow of EPA from terrestrial to aquatic ecosystems via host manipulation from the middle of August to late October, when the nematomorphs induce their terrestrial hosts to enter the water. The daily EPA intake of an endangered charr population (*Salvelinus leucomaenis japonicus*) from camel crickets and aquatic invertebrates was separately estimated by multiplying their respective EPA contents and the daily area-based consumption per the estimated charr biomass (156 g dry weight per 100 m^2^). The endangered charr population acquired 1.6–2.1 and 24.3–60.9 times more EPA from the camel crickets than from the aquatic invertebrates in T1 and T2, respectively (Fig. 1D), if evacuation rate (9) and assimilation rate were assumed to be the same for aquatic invertebrates and camel crickets. The high proportion of camel crickets in the charr diet persisted from mid-August through early November (Fig. 1E) and nearly all of the camel crickets captured in the study stream were infected (6). We conclude that host manipulation by the nematomorphs is a potential driver of the reciprocal flow of EPA between terrestrial and aquatic ecosystems. At present, the mechanisms underlying the presence of measurable amounts of EPA in camel crickets remain unresolved. Camel crickets become infected by nematomorphs through the consumption of aquatic insects that harbor nematomorph cysts (6). Therefore, they could accumulate EPA from aquatic insect prey while being exposed to nematomorph infections. Subsequent host manipulation would then drive a reciprocal flow of EPA from terrestrial to aquatic ecosystems. Integrating CSIA, which can identify the origin of assimilated EPA in animal tissues, will provide a more precise quantification of the parasite-mediated EPA fluxes between forest and stream ecosystems.

In temperate streams, aquatic invertebrate biomass, i.e. in situ EPA source, declines from spring to summer due to the seasonal emergence of adult aquatic insects (6, 10). The stream inputs of the EPA-bearing camel crickets, therefore, help compensate for this seasonal deficit of the in situ EPA availability and might contribute to supporting the nutrient demands of fish consumers during reproduction and overwintering (4). In addition, ∼2,000 species of nematomorphs are estimated to be distributed in riparian ecosystems across the globe through their associations with various arthropods, including orthopterans, coleopterans, mantids, and myriapods (11). Mermithid nematodes are also known to infect diverse terrestrial arthropods and manipulate them to enter the water (12). Therefore, the parasite-mediated EPA flow shown in this study may be a common mechanism causing the reciprocal flow of EPA in the interface of terrestrial and aquatic ecosystems. Host manipulation by parasites is ubiquitous in nature (13) but quantitative evidence of their effects on ecosystems is limited (13, 14). This study may represent one of many examples shedding light on the diverse and significant roles of manipulative parasites in natural ecosystems.

Our findings further point to the importance of examining the fluxes of organic compounds in coupled ecosystems. Since essential organic compounds often directly regulate the performance of consumers at higher trophic levels, the fluxes of these organic compounds may mediate the structure and dynamics of these ecosystems in more direct ways, such as through top-down processes. This may differ from the mechanisms by which inorganic nutrient cycling, especially nitrogen and phosphorus cycles, often regulates coupled ecosystems through bottom-up processes (15, 16). Uncovering the generality and quantitative significance of the potential reciprocal flow of PUFA and other organic compounds across ecosystems will be a crucial next step in advancing our understanding of organic nutrient fluxes and their roles in nature (1).

## Materials and methods

We first estimated daily prey consumption of camel crickets and in situ aquatic invertebrates by fish (as prey dry mass per 100 mg dry mass of fish) separately using the food consumption model (9), which incorporated a temperature-dependent gastric evacuation rate into the calculation. Mass of daily prey consumption was subsequently converted into the mass of daily area-based EPA intake per fish biomass based on the mass-based EPA of each camel cricket and aquatic invertebrate.

EPA content of camel crickets was obtained by fatty acid analysis conducted in this study (SI), whereas EPA contents of aquatic invertebrates were estimated using a meta-analysis of previously published studies conducted in rivers and streams (SI). A more in-depth account of the methods can be found in SI Appendix, and all data and codes used in this study are available in Zenodo (https://doi.org/10.5281/zenodo.15003385) (17).

Fish capture was conducted under a special collection permit issued by Nara Prefecture, which was granted upon submission of a letter of consent from a local fishery cooperative (the name is disclosed for the conservation purpose), the organization managing fisheries in the study watershed. At the time the field survey on fish consumption was conducted, the use of fish in the procedures described was not subject to mandatory review or approval by the Institutional Animal Care and Use Committee or equivalent body at Nara Women's University under the institutional and national guidelines then in effect in Japan. This determination was subsequently confirmed by the Chair of the Animal Care and Use Committee for Nara Women's University. All fish were released back into their original stream after the necessary investigations were completed.

## Supplementary Material

pgag201_Supplementary_Data

## Data Availability

Raw data and R codes used in the study are available in zenodo (https://doi.org/10.5281/zenodo.15003385).
