# Supplementary material for: Nematomorph parasites potentially drive nutritional flow from terrestrial to aquatic ecosystems
Source: PNAS Nexus. 2026 Jul 7;5(7):pgag201. doi: 10.1093/pnasnexus/pgag201 (PMC13339072; doi:10.1093/pnasnexus/pgag201)
Supplement: pgag201_Supplementary_Data [file pgag201_supplementary_data.docx]

**Supporting Information for**

**Nematomorph parasites potentially drive nutritional flow from terrestrial to aquatic ecosystems**

Ayano Medo**^a*^** and Takuya Sato**^a*^**

**^a^** Center for Ecological Research, Kyoto University, Japan

**Corresponding authors**

**Ayano Medo**: Address: 2-509-3, Hirano, Otsu, Shiga 520-2113 Japan; E-mail (1): medo.ayano.2y@kyoto-u.ac.jp; E-mail (2): medo.ayano@gmail.com; TEL: +81-77-549-8213

**Takuya Sato**: Address: 2-509-3, Hirano, Otsu, Shiga 520-2113 Japan; E-mail: [tsato@ecology.kyoto-u.ac.jp](mailto:tsato@ecology.kyoto-u.ac.jp); TEL: +81-77-549-8258

**This PDF file includes:**

Supporting text for the extended methods

SI References

## Extended Methods

The field sampling was conducted as a part of our previous study quantifying the nematomorph-mediated energy flow from forest to stream ecosystems (1), except for the collection of camel crickets and nematomorphs for the fatty acid (FA) analysis. The original data for fish stomach contents have been published (1), but they were completely re-analyzed in this study to estimate eicosapentaenoic acid (EPA) contents (mg·g⁻¹ in dry weight ) and relative EPA amount (%) in hosts and aquatic invertebrates, and EPA intake by a fish consumer. All data analyses were conducted on the R version 4.4.2 (2) at a statistical significance level of a = 0.05.

## Study site

The study was conducted in a headwater stream in an upper drainage of the Totsu River system, Kii Peninsula, Honshu, Japan (34°5’ N, 135°33’ E). The study stream was a typical temperate forest stream and was characterized by 1.14 km^2^ drainage area, 0.7 km length, 8.4% gradient, 2-5 m in width and 3.7 ± 0.7 m^3^/ sec discharge. Planted coniferous trees (*Cryptomeria japonica* and *Chamaecyparis obtusa*) and secondary-growth deciduous trees (e.g., *Fraxinus spaethiana*, *Lindera triloba*, and some species of the genus *Acer*) dominated the surrounding riparian forest (canopy cover: 70-100%). Endangered charr (Salmonidae: *Salvelinus leucomaenis japonicus*) and small numbers of minnows (Cyprinidae: *Phoxinus oxycephalus jouyi*) inhabited the study stream. Several species of nematomorphs belonging to genus Gordionus and Gordius infect camel crickets (Rhaphidophoridae: *Diestrammena elegantissima*, *D. asynamorus*, *D. tsushimensis* and *D. itoda*) and grasshoppers (Tettigoniidae: *Kinkiconocephalopsis koyasanesis* and *Psyrana japonica*), without strong host specificity (3).

## Fish sampling

Fish sampling was conducted in the study stream from August to October, when nematomorphs manipulate their terrestrial hosts, in 2007 using battery-powered backpack electrofishing units operating at 300V pulsed-DC.

On each date, we collected individual fish (> 100 mm in fork length, FL, n = 4-10) every 4 h (20, 24, 4, 8, 12 and 16 h) over a 24-h period. At the time of capture, the FL (to the nearest mm) and weight (to the nearest 0.1 g) of each fish were measured, and the stomach contents were pumped and preserved in 70% ethanol. To minimize effects of multiple captures by electrofishing on fish behavior and feeding rates, we did not sample the same 20 m reach more than once during a sampling date.

Fish stomach contents were identified to species for terrestrial orthopterans and to order for other terrestrial invertebrates and aquatic invertebrates. Individual prey was measured to the nearest 0.01 mm using calipers, dried at 60 °C for 24 h, and weighed to the nearest 0.01 mg.

## Estimation of EPA contents of aquatic invertebrates

EPA data for aquatic invertebrates in river and stream food webs were collected from the databases Web of Science and Google Scholar. The search was conducted using the following keywords: “fatty acid”, and “river” or “stream”, combined with “invertebrate”. All relevant studies published before March 2025 were included. Data were selected based on the following four criteria: (1) Experimental data from laboratory or field manipulation were excluded; (2) The study reported EPA content in units of mg g^-1^ or µg mg^-1^; (3) The study focused on rivers or streams from temperate and subarctic regions; and (4) EPA content data were categorized at a taxonomic level finer than order. Following these criteria, we excluded papers only reporting the EPA contents of species that is unusual prey for salmonid fishes (three papers were excluded on this criterion). We obtained 58 EPA content records from five studies (4-8), including four major orders in temperate rivers and streams, i.e., Ephemeroptera, Plecoptera, Trichoptera, and Amphipoda.

To estimate the probability distribution of EPA contents by synthesizing multiple studies, we applied a Bayesian random-effects meta-analysis using the *brms* package in R (Bürkner, 2017). A Bayesian framework is particularly advantageous in meta-analyses based on a limited number of studies (Röver, 2020). First, we summarized the dataset by calculating the mean, standard deviation (SD), and sample size for each study. If the EPA content was reported at a finer taxonomic resolution than order or by factors such as season and site, they were analyzed as separate data sources. The data sources with small sample sizes (n < 1) were removed from the posterior distribution estimates (nine data sources were removed in this process), resulting in 49 available data sources. A Weighted Gaussian Model was applied to the response variable (EPA content) to account for the uncertainty of measurements using standard errors. The model included 'data source' as a random effect with a Half-Cauchy prior, which was defined as μ = 0 and σ = 0.3. The intercept was set as a normal prior defined by the mean and SD of EPA content. Posterior distributions were estimated using Markov Chain Monte Carlo (MCMC) sampling with the following settings: 10000 iterations, 2000 warm-up iterations, 4 chains, and 4 cores. Model convergence was evaluated using R-hat diagnostics, with all values confirming convergence ($\hat{R}$ < 1.01). Additionally, trace plots and posterior predictive checks were examined using *plot* and *pp_check* functions in the *brms* package in R to ensure adequate model performance. Posterior distributions were estimated at two levels: (i) Order level (i.e., Ephemeroptera, Plecoptera, Trichoptera, and Amphipoda) and (ii) Arthropoda level, including all aquatic invertebrate taxa. The posterior distributions for aquatic invertebrates by Order level were visualized as probability density plots (Figure 1B), while that for Arthropoda level was used to estimate the area-based daily EPA intake by fish. The implemented models using *brms* have been available on (9).

## Estimation of EPA contents of camel crickets

*Fatty acid analysis*

Camel crickets (n = 10) were captured using sweep nets and bait traps [plastic case (L × W × H: 100 × 230 × 90 mm) with one-way entrance (23 mm bore diameter)] from October 25 to 28, 2024. Captured crickets were individually kept at approximately 4 ºC in a cooler bag and transported alive to the laboratory, where they were stored at approximately -20 ºC in a freezer until FA analysis. Before the FA analysis, all individuals were morphologically identified to species and their gut were removed (10).

All FA samples of crickets were freeze-dried for 24 hours and crushed using scissors. A dry mass of 20 mg from whole bodies were used for lipid extractions following the Bligh–Dyer method (11). The chloroform layer was separated from the methanol and dried to a constant weight under a stream of nitrogen to obtain lipids. Fatty acid methyl esters (FAMEs) were prepared by an acid-catalyzed one-step process following the method of Ichihara & Fukubayashi (12). In this process, the lipids were methylated using an HCl–methanol reagent (5–10%, w/w; Tokyo Chemical Industry, Tokyo, Japan) at 95 °C for 3 h. FAME samples were analyzed quantitatively and qualitatively by gas chromatography (GC) coupled with a mass spectrometer (MS; 5977B GC/MSD, Agilent Technologies, CA, USA). The GC-MS system was equipped with an Omegawax capillary column (30 m × 0.25 mm, i.d., 0.25-µm film thickness; Supelco, PA, USA) and operated under the following temperature gradient: the initial column temperature was set at 140 °C and held for 2.5 minutes, followed by an increase of 4 °C/min until reaching 240 °C, which was then maintained for 15 minutes, resulting in a total runtime of 42.5 minutes. All samples were injected in split mode (97:1). Helium was used as carrier gases for GC-MS. FAME were identified by comparing retention times with those of analytical standard mixtures (37-component FAME mix, Supelco 47885-U) and by analyzing spectrographic patterns with mass spectral libraries (NIST107.LIB and NIST21.LIB). The content of EPA was quantified using calibration curves based on known standard concentrations (cis-5,8,11,14,17-Eicosapentaenoic acid methyl ester, Supelco CRM47571).

*Estimation procedure*

We applied a Bayesian random sampling approach to estimate EPA content (mg g^-1^) of camel crickets using the *brms* package (13). The dataset was summarized by the mean, SD, and sample size, and a Weighted Gaussian Model was applied to the response variable (Y), where Y represents EPA contents. Measurement uncertainty was incorporated by including standard errors, and the model assumed an intercept-only structure with a normal prior based on the mean and SD of Y. Posterior distribution of EPA content of the camel crickets was estimated using the same MCMC settings as described above for aquatic invertebrates. The posterior distribution was then visualized as probability density plots in Figure 1B and was also used to estimate the area-based daily EPA intake by fish.

## Estimation of body mass and EPA content per individual prey ingested by fish for each prey category

Using the same Bayesian random sampling approach described above for camel crickets, posterior distributions were estimated for the body mass of crickets and aquatic invertebrates ingested by fish. Prior data sets of body mass (mg dry weight) of crickets and aquatic invertebrates ingested by fish were respectively obtained based on published data (1). Estimates of EPA content per individual prey for each taxon were calculated by multiplying 1000 randomly drawn samples from the posterior distributions of EPA content and body mass. The final estimates are presented as mean values (point estimate) with 95% credible interval (CI) in Figure 1C.

## The area-based daily EPA intake by fish

We estimated area-based daily EPA intake by the charr population from two distinct prey sources, i.e., camel crickets (a major host of the nematomorphs) and *in-situ* aquatic invertebrate prey. We first estimated daily prey consumption of camel crickets and aquatic invertebrates by fish (as prey dry mass per 100 mg dry mass of fish) separately using the food consumption model (14), which incorporated a temperature-dependent gastric evacuation rate into the calculation as follows. Mass of daily prey consumption was subsequently converted into the mass of daily EPA intake based on the mass-based EPA of each camel crickets and aquatic invertebrates. Consumption and EPA data were aggregated to estimate the area-based EPA intake by the charr population, assuming mean daily EPA intake per fish and fish biomass were constant during the study period.

1. We estimated the daily prey consumption (dry mass per 100 mg dry mass of fish) separately for camel crickets and aquatic invertebrates using the prey consumption model (14) (see also, its application to the same charr population (1)): *C_t_* = (*S_t_* – *S_0_* *e^-Rt^*) *R_t_*/ (1 – *e^-Rt^*), where *C* is the dry mass (mg) of prey consumed per 100 mg dry mass of trout over *t* hours, *S_0_* and *S_1_* are respectively the mean dry masses (mg) of prey in the stomach per 100 mg dry mass of fish at the beginning and the end of the sample period (*t* hours long), *e* is the exponent of the natural logarithm, and *R* is the rate of gastric evacuation per time.
2. The gastric evacuation rate (*R*) was estimated using the following equation (15): *R* = e^0.224^*^T^*^-5.44^ , where *T* is the average water temperature during each of the six sampling periods on the day of sampling.
3. The dry mass of captured fish was estimated using the formula developed for the same charr population (S1):

*W_dry_* = 0.2041*W_wet_* - 0.2232 (n = 9, *r^2^* = 0.96, error = 7.2 ± 5.1%),

where *W_dry_* and *W_wet_* are the dry and wet mass of fish, respectively.

1. The *S_t_* and *S_0_* were estimated using the Bayesian random sampling models for camel crickets and aquatic invertebrates separately. The posterior distributions were estimated using prior datasets for *S_t_* and *S_0_* that were obtained by summarizing empirical data (mean, SD, and sample size) at five periods (i.e., 16:00–20:00, 20:00–0:00, 0:00–4:00, 4:00–8:00, and 8:00–12:00). The 1000 posterior samples were drawn from these distributions, applied to the prey consumption model for each period, and converted to a daily consumption rate by summing the results from all periods. The daily consumption rate of the camel crickets was multiplied by 0.95 to conservatively estimate the mass of camel crickets confirmed to be infected by the nematomorphs.
2. Area-based daily EPA intake was calculated as:

Area-based daily EPA intake = daily consumption rate (g) * EPA content (mg g^-1^) * fish mass / 100 m^2^

The daily consumption rate (g) for aquatic invertebrates was calculated by order, based on the proportion of each order in the charr diet (1). EPA contents (mg g⁻¹) for each order (i.e., Ephemeroptera, Plecoptera, Trichoptera, and Amphipoda) were obtained using Bayesian random sampling models (n = 1000). For other prey categories (i.e., Diptera, Neuroptera, and Coleoptera) that were present in the charr diet but lacked posterior distributions in the meta-analysis, EPA contents (mg g⁻¹) were estimated via Bayesian random sampling (n = 1000) using the integrated posterior distribution for aquatic invertebrates. Similarly, EPA contents (mg g⁻¹) of camel crickets were obtained via Bayesian random sampling models (n = 1000). Fish biomass per 100 m^2^ at the study site was set at 155529.0, based on our previous survey (1). The results of area-based daily EPA intake were presented as mean values with 95% CIs for crickets and aquatic invertebrates and for T1 and T2 (Figure 1D). Moreover, to validate the persistence of EPA flux from camel crickets to charr during the study period, we analyzed the seasonal variation in the proportion of charr that ingested camel crickets (Figure 1E).

## Effects of nematomorph infection on EPA content of camel crickets

*EPA content in nematomorphs*

We collected six free-living nematomorphs belonging to the genera Gordius and Gordionus, which parasitize camel crickets, in an artificial pond near the Misogawa Dam in the upper Kiso River drainage system in Nagano Prefecture, central Honshu, Japan (35° 98′ 74′′ N, 137° 77′ 89′′ E) in 2024, as well as three in a headwater stream in an upper drainage of the Totsu River system in 2025 (1). We used a hand net to capture those worms swimming and/or locating on the water bottom. The captured specimens were stored at -20 ºC until the following fatty acid analysis.

Fatty acid analyses of nematomorphs were conducted using the same protocol as for camel crickets. FAMEs were obtained from each individual without pooling the specimens. A total of seven fatty acids were identified. The fatty acid composition of nematomorphs was dominated by 18:1n-9 (53.30 ± 12.13% at the Misogawa Dam; 42.98 ± 5.69% at the Totsu River), 10:0 (28.38 ± 14.71% at the Misogawa Dam; 38.44 ± 6.34% at the Totsu River), and 12:0 (11.55 ± 1.87% at the Misogawa Dam; 10.44 ± 2.28% at the Totsu River), together accounting for approximately 90% of the total fatty acids. In this study, n-3 PUFA, including EPA, were below the detection limit in GC-MS.

*Relative EPA amount in infected and non-infected camel crickets*

To test whether nematomorph infection influences the EPA levels of camel crickets, fatty acid compositions were compared between infected and non-infected camel crickets collected from two sites: the Misogawa Dam and a tributary of the Kiso River in Nagano Prefecture, central Honshu, Japan (35° 83′ 30′′ N, 137° 68′ 67′′ E) in August 2025. At each site, five infected and five non-infected camel crickets were collected, resulting in a total of 20 individuals. The camel cricket collection and FA analysis followed the same protocol as described above. The relative EPA amount (%) of infected and non-infected camel crickets was 6.12±2.18% and 5.08±1.36% for the Misogawa Dam, and 6.91±2.71% and 5.52±2.04% for the Kiso River, respectively. The Mann-Whitney U test revealed no significant differences in relative EPA amount between infected and non-infected camel crickets (W = 16 and p-value = 0.5309 for the Misogawa Dam; W = 17 and p-value = 0.4034 for the Kiso River).

Overall, this study provides new insights into EPA fluxes mediated by water-entry behavior of terrestrial invertebrates induced by nematomorph parasites. Given the infection process, camel crickets are likely to accumulate EPA through the consumption of emergent aquatic invertebrates. Meanwhile, there is also a possibility that terrestrial invertebrates biosynthesize EPA from terrestrial-derived precursors, α-linolenic acid (16). To elucidate EPA flows involving both dietary intake and biosynthesis, further studies should explore metabolic genes and test the conversion of ^13^C- and/or ^2^H-labeled precursors into EPA in camel crickets. In addition, compound-specific stable isotope analyses will help identify the origin of EPA assimilated in fish as well as invertebrate tissues, and evaluate the relative contributions of terrestrial and aquatic invertebrates to the EPA intake of stream fish, thereby improving the quantification of EPA fluxes.

**SI References**

1. T. Sato *et al.*, Nematomorph parasites drive energy flow through a riparian ecosystem. *Ecology* **92**, 201-207 (2011).

2. R Core Team., R: A Language and Environment for Statistical Computing. R foundation for statistical computing, Vienna, Austria. *URL* [*http://www.R-project.org/*](http://www.R-project.org/)*.* (2024).

3. T. Sato, K. Watanabe, S. Tamotsu, A. Ichikawa, A. Schmidt-Rhaesa, Diversity of nematomorph and cohabiting nematode parasites in riparian ecosystems around the Kii Peninsula, Japan. *Can. J. Zool.* **90**, 829-838 (2012).

4. P. Kesti *et al.*, Water quality shapes the community structure of zoobenthos in rivers but only has a minor effect on the fatty acid composition of zoobenthos and salmonids. *River Res. Appl.* **40**, 436-451 (2024).

5. T. Labed-Veydert, A. Bec, F. Perrière, C. Desvilettes, Utilization of basal resources in a forested headwater stream: a combined stable isotope and fatty acid approach. *Aquat. Sci.* **85**, 13 (2023).

6. T. Labed-Veydert, A.-M. Koussoroplis, A. Bec, C. Desvilettes, Early spring food resources and the trophic structure of macroinvertebrates in a small headwater stream as revealed by bulk and fatty acid stable isotope analysis. *Hydrobiologia* **848**, 5147-5167 (2021).

7. J. H. Larson, W. B. Richardson, J. M. Vallazza, L. A. Bartsch, M. R. Bartsch, Using a gradient in food quality to infer drivers of fatty acid content in two filter-feeding aquatic consumers. *Aquat. Sci.* **79**, 855-865 (2017).

8. O. N. Makhutova, Y. O. Mashonskaya, E. V. Borisova, N. I. Kislitsina, S. P. Shulepina, Fatty acid flux disparities between aquatic and terrestrial ecosystems in a shaded river can alter the nutritional content of consumer resources. *Aquat. Sci.* **87**, 39 (2025).

9. A. Medo, T. Sato, Data archive for: Nematomorph parasites potentially drive nutritional flow from terrestrial to aquatic ecosystems [Data set]. . *Zenodo* <https://doi.org/10.5281/zenodo.15003385> (2025).

10. R. Machida, *The Standard of Polyneoptera in Japan* (Tosho Printing Co., Ltd., Tokyo, Japan, ed. Orthopterological Society of Japan, 2016).

11. E. G. Bligh, W. J. Dyer, A rapid method of total lipid extraction and purification. *Canadian journal of biochemistry and physiology* **37**, 911-917 (1959).

12. K. i. Ichihara, Y. Fukubayashi, Preparation of fatty acid methyl esters for gas-liquid chromatography [S]. *J. Lipid Res.* **51**, 635-640 (2010).

13. P.-C. Bürkner, brms: An R package for Bayesian multilevel models using Stan. *Journal of statistical software* **80**, 1-28 (2017).

14. J. Elliott, L. Persson, The estimation of daily rates of food consumption for fish. *The journal of animal ecology*, 977-991 (1978).

15. J. Elliott, Rates of gastric evacuation in brown trout, Salmo trutta L. *Freshwat. Biol.* **2**, 1-18 (1972).

16. C. W. Twining *et al.*, The evolutionary ecology of fatty‐acid variation: Implications for consumer adaptation and diversification. *Ecol. Lett.* **24**, 1709-1731 (2021).
